# P10-09 Acceptability of technology-based physical activity interventions in obese females: a qualitative study

**DOI:** 10.1093/eurpub/ckac095.148

**Published:** 2022-08-29

**Authors:** Pierre Thérouanne, Meggy Hayotte, Florent Halgand, Fabienne d'Arripe-Longueville

**Affiliations:** 1 LAPCOS, Université Côte d'Azur, Nice, France; 2 Université Côte d'Azur, LAMHESS, France

**Keywords:** Obesity, physical activity, acceptability, information and communication technology

## Abstract

**Background:**

The effectiveness of technology-based physical activity interventions (TbPAI) has recently been shown for obese women (Cotie et al., 2018). However, the acceptability of TbPAI has often been considered through the measure of satisfaction, and not through the theoretical concepts of acceptability. To our knowledge, TbPAI acceptability has never been explored in bariatric surgery. The purpose of this study was to explore in-depth the facilitators of and barriers to TbPAI acceptability according to the theoretical concepts of the UTAUT2 model (Venkatesh et al., 2012).

**Methods:**

Twenty-six women with a mean age of 32.9 (*SD*=5.5) and a BMI of 30.1 (*SD*=6.5) were interviewed at least 6 months after bariatric surgery. Participants were selected in a panel of obese women who completed measures of acceptability (Hayotte et al., 2020) for each TbPAI, and expressed preference in using at least one of the technologies (10 active video games, 10 mobile application, 6 videoconferencing). Interviews were audio-recorded, transcribed verbatim, and analysed using thematic content analysis. Ethical approval was gained by local committee, and informed consent were obtained from the participants before data collection.

**Results:**

Autonomy, monitoring and feedback were identified as facilitators of mobile application acceptability. Lack of external regulation was perceived as a barrier. Regarding the acceptability of active video games, the playful dimension was perceived as a facilitator, despite several participants showing resistance to any game experience. For videoconferencing, individualization of practice was perceived as a facilitator, whereas appointment constraints were seen as a barrier.

**Conclusions:**

These preliminary results highlighted areas to be considered for the dissemination of these TbPAI in a healthcare setting. These results will also help to better individualize TbPAI counselling according to the barriers and facilitators identified.

